# Combination Effects of Metformin and a Mixture of Lemon Balm and Dandelion on High-Fat Diet-Induced Metabolic Alterations in Mice

**DOI:** 10.3390/antiox11030580

**Published:** 2022-03-18

**Authors:** Jae Young Choi, Tae-Woo Jang, Phil Hyun Song, Seong Hoon Choi, Sae-Kwang Ku, Chang-Hyun Song

**Affiliations:** 1 Department of Urology, College of Medicine, Yeungnam University, Daegu 42415, Korea; urocjy@ynu.ac.kr (J.Y.C.); sph04@yu.ac.kr (P.H.S.); 2 Department of Anatomy and Histology, College of Korean Medicine, Daegu Haany University, Gyeongsan 38610, Korea; wkdxodn12345p@dhu.ac.kr

**Keywords:** obesity, T2DM, NAFLD, dyslipidemia, kidney, HFD, metformin, combination, lemon balm, dandelion

## Abstract

Metformin, the first-line drug for type 2 diabetes mellitus (T2DM), has additional effects on improvements of nonalcoholic fatty liver disease (NAFLD); however, there are no treatments for both T2DM and NAFLD. Previous studies have shown hepatoprotective effects of a mixture of lemon balm and dandelion (LD) through its antioxidant and anti-steatosis properties. Thus, combination effects of metformin and LD were examined in a high-fat diet (HFD)-induced metabolic disease mouse model. The model received an oral administration of distilled water, monotherapies of metformin and LD, or a metformin combination with LD for 12 weeks. The HFD-induced weight gain and body fat deposition were reduced more by the combination than either monotherapy. Blood parameters for NAFLD (i.e., alanine aminotransferase and triglyceride), T2DM (i.e., glucose and insulin), and renal functions (i.e., blood urea nitrogen and creatinine) were reduced in the combination. The combination further enhanced hepatic antioxidant activities, and improved insulin resistance via the AMP-activated protein kinase and lipid metabolism pathways. Histopathological analyses revealed that the metformin combination ameliorated the hepatic hypertrophy/steatosis, pancreatic endocrine/exocrine alteration, fat tissue hypertrophy, and renal steatosis, more than either monotherapy. These results suggest that metformin combined with LD can be promising for preventing and treating metabolic diseases involving insulin resistance.

## 1. Introduction

Obesity is the most prevalent metabolic disorder caused by a high-calorie diet intake and physical inactivity, and the rising global prevalence is an emerging public health concern [1]. A significant correlation has been observed between the development of type 2 diabetes mellitus (T2DM), nonalcoholic fatty liver disease (NAFLD), and dyslipidemia [2]. Fat tissue, particularly white adipose tissue, plays a central role not only in storing the excess calories as triglycerides, but also in releasing various hormones (i.e., leptin and adiponectin) [3]. However, overwhelming the storage capacity induces altered endocrine functions in the fat tissue and then ectopic fat disposition, leading to lipotoxic metabolic stress [4]. It progresses to systemic low-grade inflammation and metabolic dysfunction in multiple organs, including the pancreas, liver, and skeletal muscle, which cooperatively promote insulin resistance and the development of T2DM, NAFLD, and dyslipidemia [3]. Over time, hyperglycemia-induced cellular oxidative stress and inflammation impair insulin secretion and β-cell function [1]. Particularly, T2DM is a major risk factor for the development of NAFLD: the global prevalence of T2DM among patients with NAFLD is 22.5%, while the prevalence of NAFLD among patients with T2DM is 55.5% [5]. Further, increasing evidence has shown that the severity of NAFLD connects to an increased risk of chronic kidney disease [6]. Thus, there is an unmet need for comprehensive therapeutic strategies for the relevant metabolic diseases that share common mechanisms linked by metabolic alterations and insulin resistance.

Metformin, an AMP-activated protein kinase (AMPK) activator, is a biguanide drug, which is recommended as the first-line treatment for T2DM [7]. It not only has a glucose-reducing effect by inhibiting hepatic gluconeogenesis, but has also been reported to have additional inhibitory effects on weight gain and inflammatory progression [8]. Although there is no approved medication for treating NAFLD, recent studies have shown that metformin can be effective in treating obesity and NAFLD by improving insulin resistance and hepatic triglyceride accumulation [9,10]. However, metformin has limited efficacy in improving the histopathological changes in NAFLD, and the clinical evidence is still lacking [11,12]. Further, metformin is contraindicated in T2DM patients with hepatic and renal dysfunction or congestive heart failure because of concerns about lactic acidosis [13,14]. Above this, peroxisome proliferator activated receptor (PPAR)γ agonist (i.e., piogliazone) has shown therapeutic potentials in patients with NAFLD, particularly nonalcoholic steatohepatitis (NASH), or T2DM, and other treatments with vitamin E, statins, or silymarin are also reported to contribute to improvement in NAFLD; however, these treatments have side effects or little evidence to support their clinical uses in NAFLD [15].

Common dandelion (*Taraxacum officinale* [L.] Weber ex F. H. Wigg) is a traditional herb used for treating various diseases, including diabetes and liver disease, because of the diverse pharmacological effects including antioxidant, anti-inflammatory, diuretic, antidiabetic, and anti-hyperglycemic properties [16]. Indeed, dandelion has shown improvements in carbon tetrachloride (CCL4)-induced liver damages [17], high-fat diet (HFD)-induced NAFLD [18], and streptozotocin-induced diabetes [19]. Lemon balm (*Melissa officinalis* L.) is also a traditional herb, involved in treating hyperglycemia, hyperlipidemia, and insulin resistance-related metabolic alterations [20,21]. We previously reported that a mixture consisting of dandelion and lemon balm at a ratio of 2:1 (*w*/*w*) has hepatoprotective effects on ethanol- and CCL4-induced liver damage by enhancing the antioxidant and anti-inflammatory properties via activation of the nuclear factor (erythroid-derived 2)-like (NRF)2 pathway, which are greater than those of monotherapies of dandelion and lemon balm alone [22,23]. Furthermore, the mixture downregulated the expressions of hepatic lipogenesis-related genes and upregulated the expressions of fatty acid oxidation genes. It suggests that the mixture can be promising as a novel hepatoprotective agent or medicinal food ingredient for NAFLD and T2DM. Thus, we examined the effects of a combination of metformin and the mixture of dandelion and lemon balm on HFD-induced metabolic alterations in multi-systems of liver, pancreas, fat tissues, and kidney.

## 2. Materials and Methods

### 2.1. Animal Model

All animal experiments were conducted according to the national regulations of the usage and welfare of laboratory animals, and approved by the Institutional Animal Care and Use Committee in Daegu Haany University (Gyeongsan, Korea, Approval No. DHU2020-054). Six-week-old female SPF/VAF CrljOri:CD1 (ICR) mice were obtained from Orient Bio Inc. (Seongnam, Korea). They were housed five per polycarbonate cage in a temperature (20–25 °C) and humidity (40–45%)-controlled room, with a light/dark cycle of 12/12 h. Feed and water were supplied free to access. After a week of acclimatization, the mice with similar body weights were divided into one normal-fat diet group with Purinafeed (Seongnam, Korea) and six high-fat diet model groups with Rodent Diet with 45 Kcal% (#D12451, Research Diet, New Brunswick, NJ, USA) (*n* = 8/group). The mice that adapted to the high-fat diet for a week were regrouped based on their body weights. All animals were fasted overnight prior to the initial and last treatments to avoid the diet effects, and the body weights were measured daily.

### 2.2. Treatments

Leaf extracts of lemon balm (LB) and dandelion (DL) were supplied by Evear Extraction (Coutures, France). The extracts were dissolved in distilled water (DW) and prepared as a mixture of LB and DL at 2:1 (*w*/*w*) (LD), as described previously [22,23]. Metformin hydrochloride (Wako, Osaka, Japan) was used at 250 mg/kg based on a previous animal study [24]. Six high-fat diet model groups received an oral co-administration of DW plus DW (HFD control), or DW plus metformin (Met) or LD at 200 mg/kg (LD (H)), referring 0.97 g in human adults of 60 kg using the following formula: (200 mg/kg × 60 kg × 0.081 (as a constant value for body surface area to human)), for the monotherapy, or metformin plus LD at 200, 100, and 50 mg/kg (Met + LD(H), Met + LD(M), and Met + LD(L), respectively) for the metformin combination groups. The Intact group received an oral co-administration of DW plus DW. The co-administration was performed in a volume of 10 mL/kg at 1 h intervals for 12 weeks, based on the pharmacokinetic analyses. After all treatments, blood was collected under anesthesia with 2–3% isoflurane in a mixture of 70% N_2_O and 28.5% O_2_, and the mice were euthanized using a CO_2_ gas. Then, the liver, pancreas, left periovarian/abdominal fat pads, and left kidney were sampled, and the organ weights were measured. The blood and tissue samples were subjected to biochemical and histopathological analyses.

### 2.3. Assessment of Food Consumption, Lipid Excretion, and Body Fat Deposition

For daily food consumption, diets of 150 g were supplied to each cage, and daily intake was measured every week. Lipid excretion was assessed in feces sampled 8 h after the last treatment, using the Triglyceride Colorimetric Assay Kit (#10010303, Cayman, Ann Arbor, MI, USA) and the Total Cholesterol Assay Kit (colorimetric) (#STA-384, Cell Biolabs, San Diego, CA, USA), according to the manufacturers’ protocols. The levels were examined in duplicate using a plate reader (Tecan, Männedorf, Switzerland). Fat density was assessed in total body and the abdominal regions using dual-energy X-ray absorptiometry (DEXA; Medikors, Seongnam, Korea).

### 2.4. Blood Biochemistry

A small volume of whole blood was analyzed for the blood glycated hemoglobin (HbA1c) using EasyA1c (Infopia Co., Anyang, Korea). The other samples were collected in sodium fluoride glucose vacuum tubes (Becton Dickinson, Franklin Lakes, NJ, USA) to assess the blood glucose level, and clotting-activated serum tubes for serum levels of alkaline phosphatase (ALP), alanine aminotransferase (ALT), aspartate aminotransferase (AST), lactate dehydrogenase (LDH), gamma-glutamyltransferase (GGT), blood urea nitrogen (BUN), creatinine, triglyceride, total cholesterol, and low-/high-density lipoprotein cholesterol (LDL/HDL). The levels were assessed using Dri-Chem NX500i (Fuji Medical System Co., Ltd., Tokyo, Japan). Insulin level was measured using a mouse Insulin ELISA Kit (#80-INSMS-E01; Alpco Diagnostics, Windham, NH, USA).

### 2.5. Hepatic Antioxidant Activities

Some liver tissue (about 0.3 g) was prepared as 10% homogenates in ice-cold 0.01 M Tris-HCl (pH 7.4) for assessing the antioxidant activities, as described previously [24]. Briefly, the level of malondialdehyde (MDA) for lipid peroxidation was assessed using the thiobarbituric acid assay at an absorbance of 525 nm. The glutathione (GSH) level was measured at 412 nm using 2-nitrobenzoic acid. For catalase activity, the amount of catalase required to decompose 1 nM of H_2_O_2_ (pH 7.8) per min at 25 °C was measured at 240 nm. Superoxide dismutase (SOD) activity was estimated as the generation of superoxide radicals produced by xanthine and xanthine oxidase which react with nitrotetrazolium blue to form formazan dye, and measured at 560 nm. One unit of SOD enzyme activity is the amount diminishing the initial absorbance of nitrotetrazolium blue by 50% for 1 min. All levels were measured using a plate reader (Tecan), and normalized to the tissue protein contents.

### 2.6. Hepatic Glucose-Regulating Enzyme Activities

Liver tissue (about 0.3 g) was homogenized as 10% (*w*/*v*) in 0.1 M triethanolamine, 0.2 M EDTA, and 2 mM dithiothreitol. The supernatant was collected after centrifuging at 1000× *g* and then at 10,000× *g* for 15 min at 4 °C, and used for assessing the hepatic enzyme activities. For glucokinase (GK) activity, the supernatant (10 μL) was incubated with the reaction buffer (0.98 mL) containing 50 mM HEPES-NaGT (pH 7.4), 100 mM KCl, 7.5 mM MgCl_2_, 2.5 mM dithioerythritol, 10 mg/mL albumin, 10 mM glucose, and 4 units of glucose-6-phosphate dehydrogenase, at 37 °C for 10 min. The reaction was initiated by incubating with 5 mM ATP (10 μL) at 37 °C for 10 min, and measured at 340 nm. For glucose-6-phosphatase (G6pase), the supernatant (5 μL) was incubated with the buffer containing 765 μL of 131.58 mM HEPES-NaGT (pH 6.5), 100 μL of 18 mM EDTA (pH 6.5), 100 μL of 265 mM glucose-6-phosphate, 10 μL of 0.2 M NADP^+^, 0.6 IU/mL of mutarotase, and 0.6 IU/mL of glucose dehydrogenase at 37 °C for 4 min, and measured at 340 nm. For phosphoenolpyruvate carboxykinase (PEPCK), the supernatant (10 μL) was mixed with the buffer (0.99 mL) containing 72.92 mM sodium HEPES (pH 7.0), 10 mM dithiothreitol, 500 mM NaHCO_3_, 10 mM MnCl_2_, 25 mM NADH, 100 mM inositol-1,4-diphosphate, 200 mM phosphoenolpyruvate, and 7.2 units of malic dehydrogenase. The activity was measured according to the reduced absorbance at 340 nm using a UV/Vis spectrophotometer (OPTIZEN POP, Mecasys, Daejeon, Korea). All the reagents were purchased from Sigma-Aldrich (St. Louis, MO, USA).

### 2.7. Reverse Transcription-Polymerase Chain Reaction (RT-PCR) Analysis

Expressions of mRNAs, acetyl-CoA carboxylase 1 (ACC1), AMP-activated protein kinase (AMPK)α1, and AMPKα2 were measured in the liver, and gene expressions of leptin, adiponectin, uncoupling protein (UCP)2, CCAAT-enhancer-binding protein (C/EBP)α, C/EBPβ, sterol-regulatory-element-binding protein 1c (SREBP1c), PPARα, PPARγ, and fatty acid synthase (FAS) were measured in the periovarian fat tissue as a predominant region of white adipose tissues, as described previously [24,25]. Total RNAs (about 5 μg) were extracted using TRIzol reagent (Invitrogen, Carlsbad, CA, USA), and the concentration and quality were determined by the CFX96^TM^ Real-Time System (Bio-Rad, Hercules, CA, USA). The contaminating DNA was removed using recombinant DNase I (Ambion, Austin, TX, USA). The RNA was reverse-transcribed using the reagent High-Capacity cDNA Reverse Transcription Kit (Applied Biosystems, Foster City, CA, USA). The PCR conditions were as follows: 10 min at 94 °C, 39 cycles of 15 s at 94 °C, 20 s at 57 °C, and 30 s at 72 °C. The expression was analyzed by the ABI Step One Plus^TM^ Sequence Detection System (Applied Biosystems), and normalized to that of GAPDH using the comparative threshold cycle method [26]. The oligonucleotide primers used for the PCR are listed in Supplementary Table S1.

### 2.8. Histopathology

Samples of the liver, pancreas, fat mass, and kidney were fixed in 10% neutral buffered formalin, paraffin-embedded, and serially sectioned at 3–4 μm. The sections were stained with hematoxylin and eosin (HE), and examined for diameter of the hepatocyte and adipocytes (in at least ten cells), thickness of fat tissues, area occupying pancreatic zymogen granules, number and diameter of pancreatic islets, and numbers of vacuolated renal tubules. A portion of the liver was dehydrated in 30% sucrose solutions, and frozen and sectioned at 20 μm. The sections were stained in oil red O, and the stained regions were examined. The histomorphometric analyses were performed using a computer-assisted automated image analyzer (*i*Solution FL ver 9.1, IMT *i*-solution Inc., Vancouver, BC, Canada) by a histopathologist blinded to the groups.

### 2.9. Immunohistochemistry

Other serial sections of the pancreas were treated with 0.3% H_2_O_2_ in methanol for 30 min, and then with horse serum blocking solution (Vector Lab., Burlingame, CA, USA., a dilution of 1:100) for 1 h. The sections were incubated with guinea pig anti-insulin (#ab7842; Abcam, Cambridge, UK, a dilution of 1:100) and rabbit anti-glucagon antibodies (#ab133195; Abcam, a dilution of 1:100) overnight at 4 °C. The next day, they were incubated with biotinylated universal secondary antibody (Vector Lab., a dilution of 1:50) for 1 h and ABC reagents (#PK-6200; Vector Lab., a dilution of 1:50) for 30 min. The immunoreactivity was visualized with the peroxidase substrate kit (#SK-4100; Vector Lab., a dilution of 1:50) for 3 min, and counterstained with hematoxylin. All sections were incubated in a humidity chamber, and rinsed in 0.01 M phosphate-buffered saline three times between each step. The cells occupying more than 20% of immunoreactivities were regarded positive, and they were counted using an *i*Solution image analyzer by a histopathologist blinded to the groups.

### 2.10. Statistical Analyses

All data were expressed as the means ± standard deviations (SDs) of ten sample sizes. Since the Kolmogorov–Smirnov tests showed a normal distribution of the variables, data were examined by one-way analysis of variance (ANOVA). Kinetic changes of body weights were examined by two-way ANOVA with main factors for the groups and the time-points measured, and the time-point was treated as a repeated measure. Based on homogeneity of variance by Levene’s test, the multiple comparison was examined by Tukey and Dunnett’s T3 post-hoc tests in cases of equal and non-equal variances, respectively. A *p*-value less than 0.05 is considered statistically significant.

## 3. Results

### 3.1. Combination Effects on Body Weight Loss

**Figure 1:**
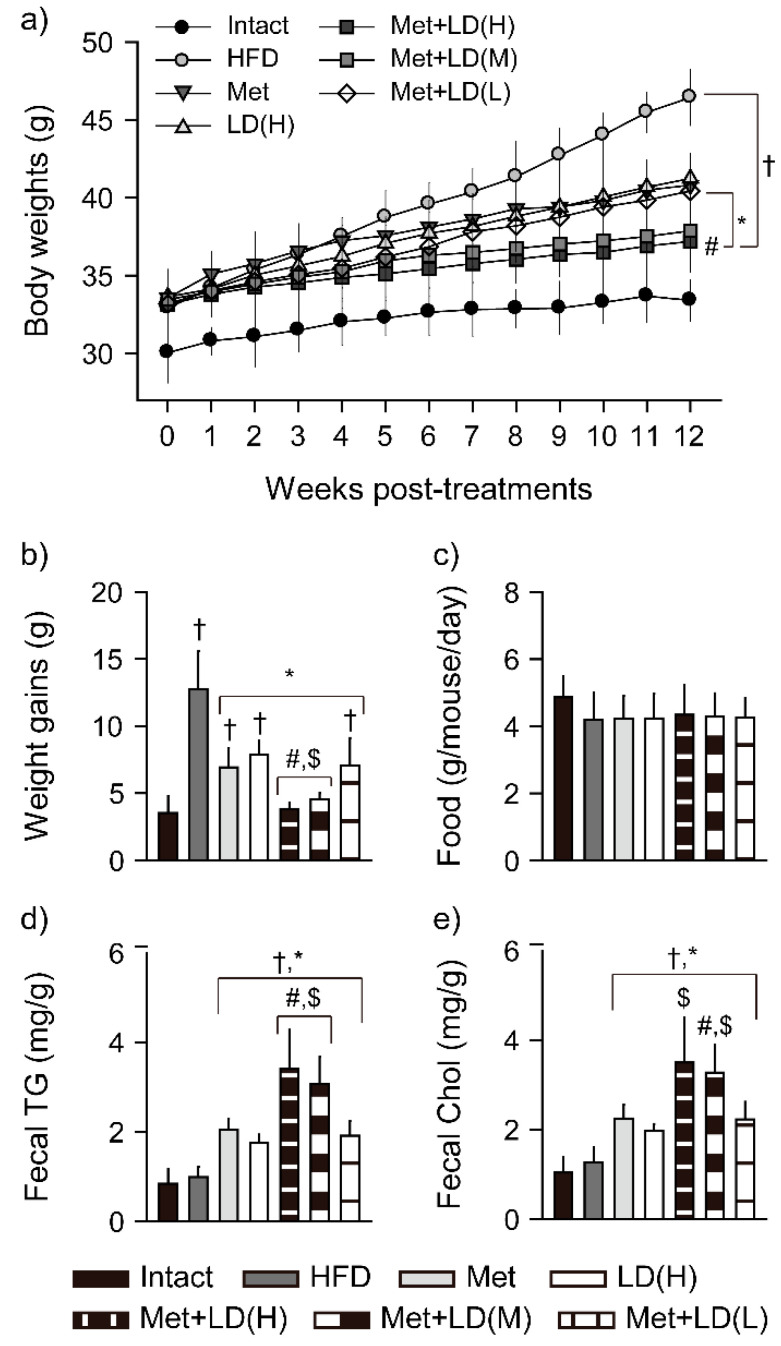
Combination effects on weight loss and energy metabolism. (**a**) Kinetic changes in body weights. (**b**) Total weight gains for 12 weeks. (**c**) Food consumption. (**d**,**e**) Fecal excretion of triglyceride (TG) and total cholesterol (Chol). Values are represented as the means ± standard deviations (SDs) (*n* = 8). †, *, #, and $: *p* < 0.05 vs. the Intact, HFD control (HFD), Met, and LD(H) groups, respectively.

Anti-obesity effects were examined by the body weight change, food consumption, and fecal lipid excretion (Figure 1). Two-way ANOVA for the kinetic body weight changes showed significant main effects for the groups and the time-points measured (*p* < 0.01, Figure 1a). The kinetic changes were increased in all of the HFD model groups vs. the Intact group (*p* < 0.01); however, they were reduced in three metformin combination groups vs. the HFD control, and further reduced in the Met + LD(H) vs. the Met group (*p* < 0.05). There were also significant interactions between the groups and the time-points (*p* < 0.01), meaning time-dependent group differences. The body weights were increased in all of the HFD models regardless of the treatments vs. the Intact group. However, compared with the HFD control, they were reduced in week 9 to 12 post-treatments in the Met and LD(H), in week 4 to 12 in the Met + LD(H), in week 5 to 12 in the Met + LD(M), and in week 8 to 12 in the Met + LD(L) (*p* < 0.05). The body weights were further reduced in week 7 to 12 and week 8 to 12 in the Met + LD(H) vs. the Met and the LD(H), respectively, and they were also reduced in week 10 to 12 in the Met + LD(M) vs. both monotherapy groups (*p* < 0.05). The total weight gains were increased in the HFD control vs. the Intact group; however, they were reduced in all treatment groups vs. the HFD control, and further reduced in the Met + LD(H) and Met + LD(M) vs. both monotherapy groups (*p* < 0.05, Figure 1b).

### 3.2. Combination Effects on Fecal Lipid Excretion

There were no differences in daily food consumption among the groups (Figure 1c); however, there were significant differences in amounts of the fecal triglycerides and total cholesterol (*p* < 0.01, Figure 1d,e). The fecal triglycerides and total cholesterol were not different between the Intact and the HFD control groups; however, they were significantly increased in all treatment groups compared with the Intact and the HFD control groups (*p* < 0.01). Further, the fecal triglyceride was excreted more in the Met + LD(H) and Met + LD(M) than both monotherapy groups, and the fecal cholesterol was higher in the Met + LD(H) than the LD(H) group and in the Met + LD(M) than both monotherapy groups (*p* < 0.05).

### 3.3. Combination Effects on Inhibition of Body Fat Deposition

**Figure 2:**
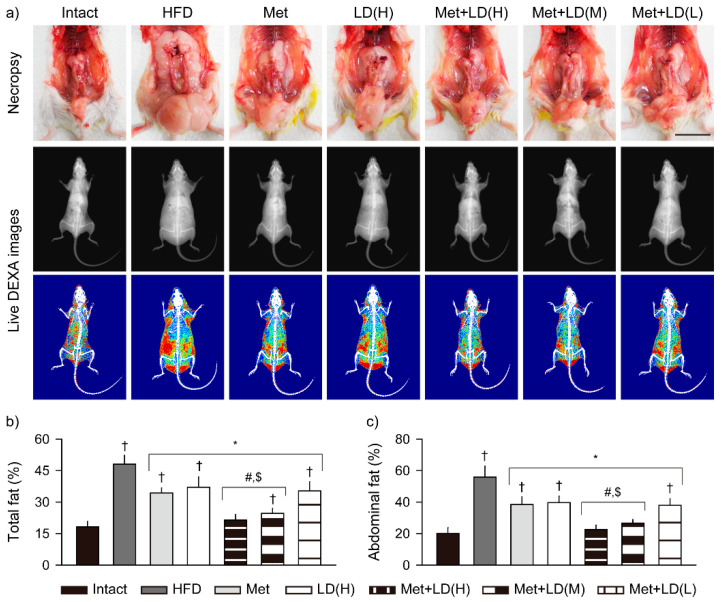
Combination effects on inhibition of body fat deposition. (**a**) Representative images in necropsy (upper) and live dual-energy X-ray absorptiometry (DEXA; lower). In live DEXA images, red, yellow, and blue indicate high-, intermediate-, and low-density fats, respectively. Scale bars = 2 cm. (**b**,**c**) Fat densities of total body and the abdominal region. Values are represented as the means ± SDs (*n* = 8). †, *, #, and $: *p* < 0.05 vs. the Intact, HFD control (HFD), Met, and LD(H) groups, respectively.

In necropsy, abdominal fat mass was evident in the HFD control; however, it tended to be reduced in the treatment groups (Figure 2a). Indeed, DEXA analyses showed significant increases in the total fat and abdominal fat densities in the HFD control vs. the Intact group (*p* < 0.01); however, the densities were reduced in all treatment groups vs. the HFD control, and further reduced in the Met + LD(H) and Met + LD(M) vs. both monotherapy groups (*p* < 0.01, Figure 2b,c).

### 3.4. Combination Effects on HFD-Induced Organ Weights

**Figure 3:**
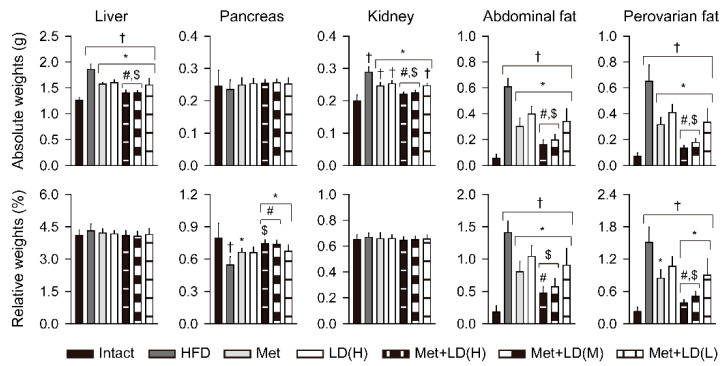
Combination effects on HFD-induced organ weights. Absolute organ weights of the liver, pancreas, kidney, and abdominal/periovarian fat mass, and the relative weights to the body weights, are represented as the means ± SDs (*n* = 8). †, *, #, and $: *p* < 0.05 vs. the Intact, HFD control (HFD), Met, and LD(H) groups, respectively.

Absolute weights of the liver, kidney, and abdominal/periovarian fat mass were increased in the HFD control vs. the Intact group (*p* < 0.01); however, they were reduced in all treatment groups vs. the HFD control, and further reduced in the Met + LD(H) and Met + LD(M) vs. both monotherapy groups (*p* < 0.05, Figure 3). For the abdominal/periovarian fat mass, the relative organ weights to the body weights also showed significant increases in the HFD control vs. the Intact group (*p* < 0.01); however, the relative weight of the abdominal fat mass was reduced in all treatment groups vs. the HFD control, and further reduced in the Met + LD(H) vs. both monotherapy groups and in the Met + LD(M) vs. the LD(H) group (*p* < 0.05). The relevant weight of the periovarian fat mas was reduced in the Met and the combination groups vs. the HFD control, and further reduced in the Met + LD(H) and Met + LD(M) vs. both monotherapy groups (*p* < 0.05). Although the absolute weight of the pancreas was not different among the groups, the relative weights were significantly reduced in the HFD control vs. the Intact group (*p* < 0.05). However, they were increased in the Met and the combination groups vs. the HFD control, and further increased in the Met + LD(H) vs. both monotherapy groups and in the Met + LD(M) vs. the LD(H) group (*p* < 0.05).

### 3.5. Combination Effects on Improvements in Blood Biochemistry

**Figure 4:**
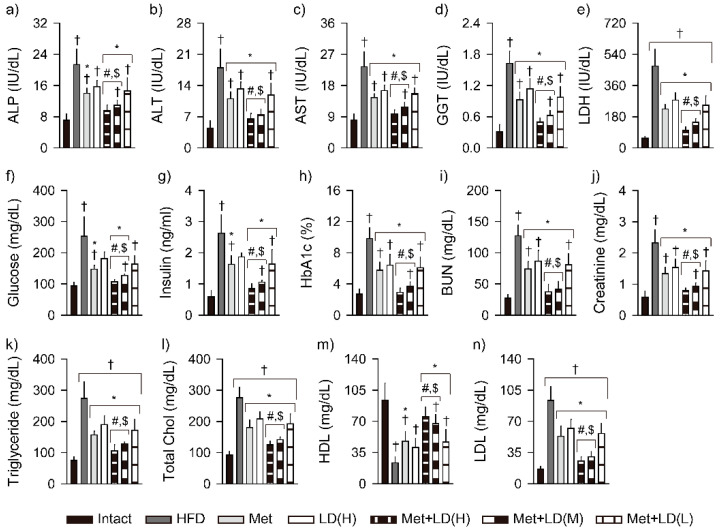
Combination effects on parameters in blood biochemistry. (**a**–**n**) Blood levels of alkaline phosphatase (ALP), alanine aminotransferase (ALT), aspartate aminotransferase (AST), gamma-glutamyltransferase (GGT), lactate dehydrogenase (LDH), glucose, insulin, blood glycated hemoglobin (HbA1c), blood urea nitrogen (BUN), creatinine, triglyceride, total cholesterol (Chol), and low-/high-density lipoprotein cholesterol (LDL/HDL). Values are represented as the means ± SDs (*n* = 8). †, *, #, and $: *p* < 0.05 vs. the Intact, HFD control (HFD), Met, and LD(H) groups, respectively.

Among the 14 parameters in blood biochemical analyses, serum HDL was reduced in the HFD control vs. the Intact group, while the others were increased (*p* < 0.01, Figure 4). The increased levels of ALT, AST, GGT, LDH, HbA1c, BUN, creatinine, triglyceride, total cholesterol, and LDL were reduced in all treatment groups, the ALP and insulin were reduced in the Met and the combination groups, and the glucose was reduced in the Met, Met + LD(H), and Met + LD(M) groups (*p* < 0.05). The levels were further reduced in the Met + LD(H) and Met + LD(M) vs. both monotherapy groups (*p* < 0.05). The level of HDL was increased in the Met and the combination groups vs. the HFD control, and further increased in the Met + LD(H) and Met + LD(M) vs. both monotherapy groups.

### 3.6. Combination Effects on Hepatic Antioxidant Defense System and Glucose Regulation

**Figure 5:**
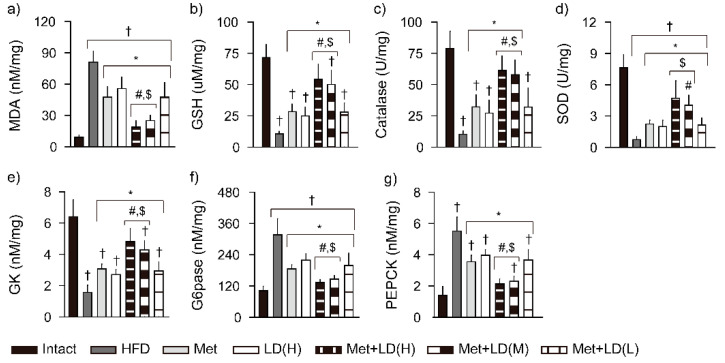
Combination effects on hepatic antioxidant defense system and glucose regulation. (**a**–**d**) Levels of malondialdehyde (MDA) and glutathione (GSH), activities of catalase and superoxide dismutase (SOD). (**e**–**g**) Glucose-regulating enzyme activities of glucokinase (GK), glucose-6-phosphatase (G6pase), and phosphoenolpyruvate carboxykinase (PEPCK). Values are represented as the means ± SDs (*n* = 8). †, *, #, and $: *p* < 0.05 vs. the Intact, HFD control (HFD), Met, and LD(H) groups, respectively.

The level of MDA for lipid peroxidation was increased in the HFD control vs. the Intact group; however, it was reduced in all treatment groups vs. the HFD control, and further reduced in the Met + LD(H) and Met + LD(M) vs. both monotherapy groups (*p* < 0.05, Figure 5). Conversely, the level of GSH and activities of catalase and SOD were reduced in the HFD control vs. the Intact group (*p* < 0.01); however, they were increased in all treatment groups vs. the HFD control, and further increased in the Met + LD(H) and Met + LD(M) vs. both monotherapy groups (*p* < 0.05). The level of GK, as a glucose sensor, was reduced in the HFD control vs. the Intact group; however, it was increased in all treatment groups vs. the HFD control, and further increased in the Met + LD(H) and Met + LD(M) vs. both monotherapy groups (*p* < 0.05). Levels of G6pase and PEPCK, as regulators of hepatic gluconeogenesis, were increased in the HFD control vs. the Intact group (*p* < 0.01); however, they were reduced in all treatment groups vs. the HFD control, and further reduced in the Met + LD(H) and Met + LD(M) vs. both monotherapy groups (*p* < 0.05).

### 3.7. Combination Effects on Gene Expressions Involved in Metabolic Alteration

**Figure 6:**
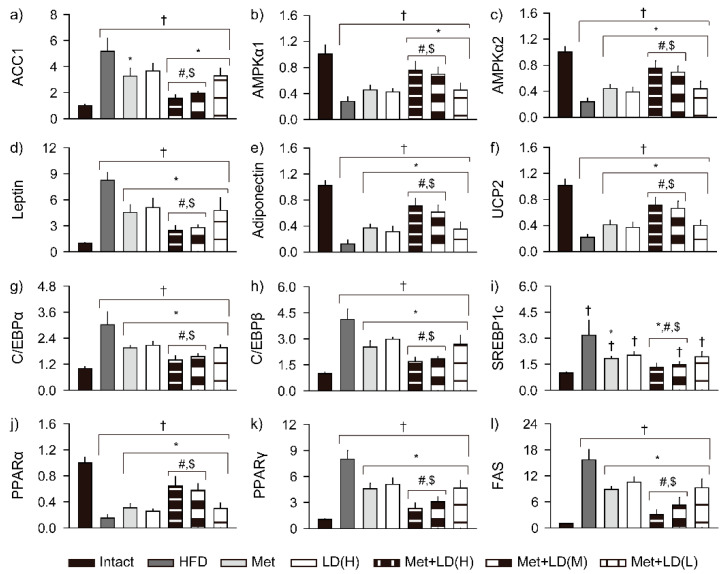
Combination effects on gene expression involved in metabolic alteration. (**a**–**c**) mRNA expressions of acetyl-CoA carboxylase 1 (ACC1), AMP-activated protein kinase (AMPK)α1, and AMPKα2 in the liver. (**d**–**l**) mRNA expressions of leptin, adiponectin, uncoupling protein (UCP)2, CCAAT-enhancer-binding protein (C/EBP)α, C/EBPβ, sterol-regulatory-element-binding protein 1c (SREBP1c), peroxisome proliferator-activated receptor (PPAR)α, PPARγ, and fatty acid synthase (FAS) in the periovarian fat tissues. Values are represented as the means ± SDs (*n* = 8). †, *, #, and $: *p* < 0.05 vs. the Intact, HFD control (HFD), Met, and LD(H) groups, respectively.

In the liver, mRNA expression of ACC1, an enzyme that catalyzes carboxylation of acetyl-CoA to malonyl-CoA for fatty acid synthesis, was upregulated in the HFD control vs. the Intact group, while expressions of AMPKα1 and AMPKα2, as master regulators of cellular energy homeostasis, were downregulated (*p* < 0.01, Figure 6). However, compared to the HFD control, the ACC1 was downregulated in the Met and the combination groups, and the AMPKα1 and the AMPKα2 were upregulated in the combination groups and in all treatment groups, respectively (*p* < 0.05). The regulation was greater in the Met + LD(H) and Met + LD(M) groups than in both monotherapy groups. In the fat tissue, lipogenesis-related gene expressions of leptin, C/EBPα, C/EBPβ, SREBP1c, PPARγ, and FAS were upregulated in the HFD control vs. the Intact group, while lipolysis-related gene expressions of adiponectin, UCP2, and PPARα were downregulated (*p* < 0.01). However, compared to the HFD control, the expressions of leptin, C/EBPα, C/EBPβ, PPARγ, and FAS were downregulated in all treatment groups, and the adiponectin, UCP2, and PPARα were upregulated (*p* < 0.05). The expression of SREBP1c was downregulated in the Met, Met + LD(H), and Met + LD(M) vs. the HFD control (*p* < 0.05). The lipid metabolism-related gene regulation in the fat tissue was also greater in the Met + LD(H) and Met + LD(M) groups than in both monotherapy groups (*p* < 0.05).

### 3.8. Combination Effects on Histopathological Changes in the Multi-System Metabolic Alteration

**Figure 7:**
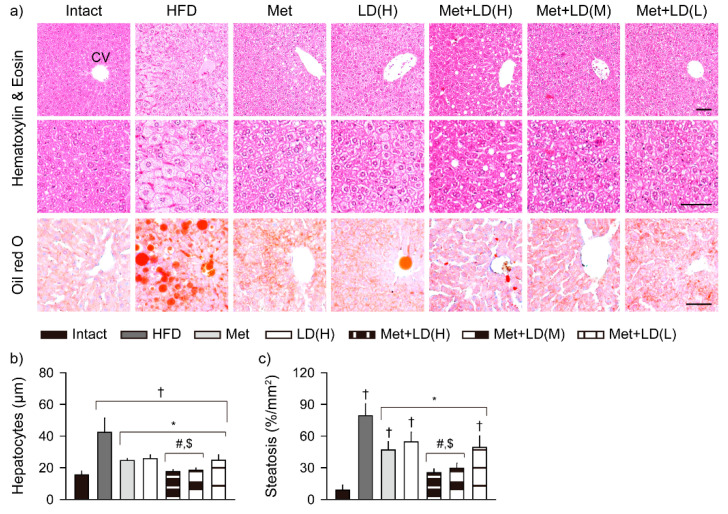
Histopathological improvements in lesions of the liver. (**a**) Representative images in stains with hematoxylin and eosin and oil red O. CV = central vein. Scale bars = 50 μm. (**b**,**c**) Sizes of the hepatocytes and steatosis areas stained with oil red O. Values are represented as the means ± SDs (*n* = 8). †, *, #, and $: *p* < 0.05 vs. the Intact, HFD control (HFD), Met, and LD(H) groups, respectively.

**Figure 8:**
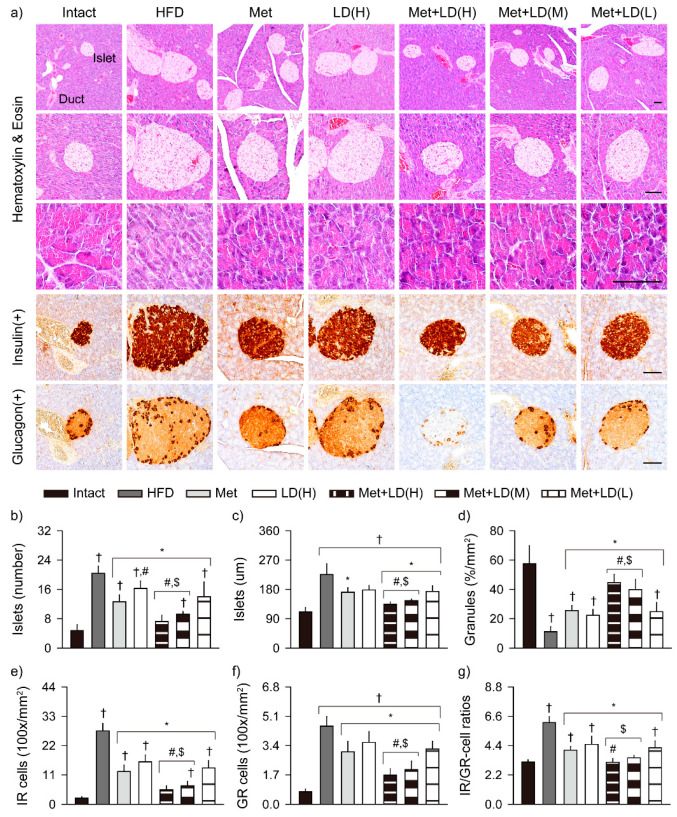
Histopathological improvements in pancreatic lesions. (**a**) Representative images in stains with hematoxylin and eosin and immuno-stains for insulin and glucagon. In hematoxylin and eosin stains, pancreatic islet and exocrine duct (upper) are indicated, and the islets and acinar regions were highly magnified (middle and lower, respectively). Scale bars = 50 μm. (**b**,**c**) Numbers and sizes of the islets. (**d**) Acinar areas containing zymogen granules. (**e**–**g**) Numbers of insulin- (IR) and glucagon-immunoreactive (GR) cells, and ratio of IR to GR cells. Values are represented as the means ± SDs (*n* = 8). †, *, #, and $: *p* < 0.05 vs. the Intact, HFD control (HFD), Met, and LD(H) groups, respectively.

**Figure 9:**
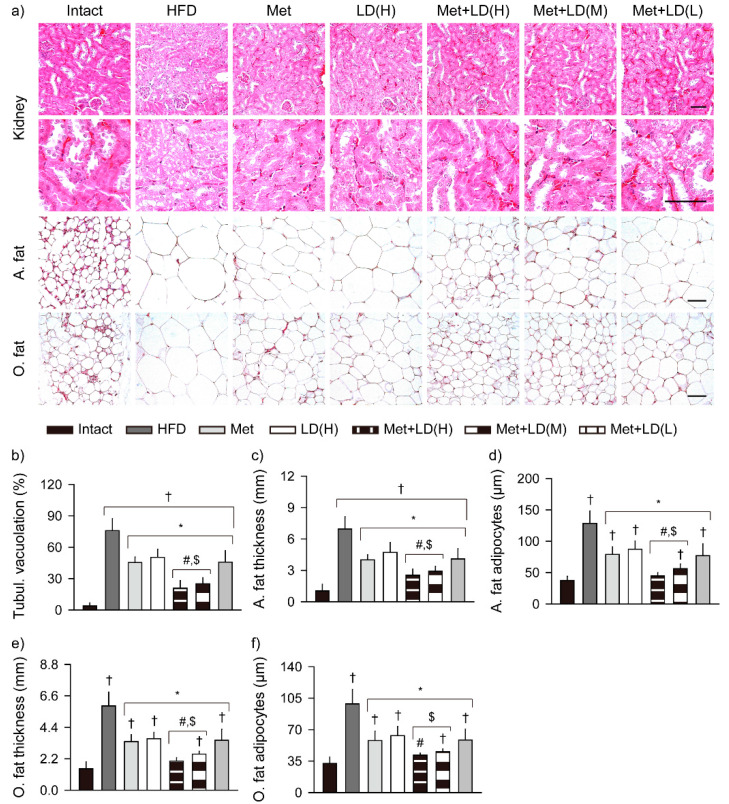
Histopathological improvements in lesions in the kidney and fat tissue. (**a**) Representative images of the kidney and abdominal (A.)/periovarian (O.) fat tissue in stains with hematoxylin and eosin. Each image was highly magnified (lower rows). Scale bars = 50 μm. (**b**) Areas with renal tubular (Tubul.) vacuolation. (**c**–**f**) Thickness of the A./O. fat tissues and sizes of the adipocytes. Values are represented as the means ± SDs (*n* = 8). †, *, #, and $: *p* < 0.05 vs. the Intact, HFD control (HFD), Met, and LD(H) groups, respectively.

The HFD control showed several histopathological abnormalities in HE stains of the multi-organs, as follows: hepatic hypertrophy with steatosis, increased/enlarged pancreatic islet, and reduced number of exocrine zymogen granules, fat tissue hypertrophy, and renal tubular vacuolation (Figure 7, Figure 8 and Figure 9). However, the changes were mild in the treatment groups, particularly in the metformin combination groups.

#### 3.8.1. Histopathological Improvements in the Liver

Histomorphometric analyses showed significant increases in the diameters of hepatocytes and areas stained with oil red O in the HFD control vs. the Intact group (*p* < 0.01); however, the sizes and areas were reduced in all treatment groups, and further reduced in Met + LD(H) and Met(M) vs. both monotherapy groups (*p* < 0.05, Figure 7).

#### 3.8.2. Histopathological Improvements in the Pancreas

The pancreatic islets were significantly increased and enlarged in the HFD control vs. the Intact group (*p* < 0.01); however, the number was reduced in all treatment groups, and the size was reduced in the metformin combination groups (*p* < 0.05, Figure 8). The insulin- and glucagon-immunoreactive cells were increased in the HFD control vs. the Intact group (*p* < 0.01); however, they were also reduced in all treatment groups vs. the HFD control. The inhibitory effects on the pancreatic islets and the immunoreactive cells were greater in the Met + LD(H) and Met(M) than both monotherapy groups (*p* < 0.05). Further, the ratio of the insulin- to glucagon-immunoreactive cells was increased in the HFD control vs. the Intact group (*p* < 0.01); however, it was reduced in all treatment groups vs. the HFD control, and further reduced in the Met + LD(H) vs. both monotherapy groups and in the Met + LD(M) vs. the LD(H) group (*p* < 0.05). The acinar area containing pancreatic zymogen granule was reduced in the HFD control vs. the Intact group (*p* < 0.01); however, it was increased in all treatment groups vs. the HFD control, and further increased in the Met + LD(H) and Met(M) vs. both monotherapy groups (*p* < 0.05).

#### 3.8.3. Histopathological Improvements in Fat Tissue and Kidney

Tubular vacuolation of the kidney was increased in the HFD control vs. the Intact group (*p* < 0.01); however, it was reduced in all treatment groups vs. the HFD control, and further reduced in the Met + LD(H) and Met + LD(M) vs. both monotherapy groups (*p* < 0.05, Figure 9). In the abdominal and periovarian fat tissues, the fat thickness and adipocyte sizes were significantly increased in the HFD control vs. the Intact group (*p* < 0.01); however, they were reduced in all treatment groups vs. the HFD control, and further reduced in the Met + LD(H) and Met + LD(M) vs. both monotherapy groups (*p* < 0.05).

## 4. Discussion

Obesity is accompanied by excessive fat accumulation, and visceral fat mass is a particularly important factor contributing to the development of metabolic diseases involved in lipid metabolic alterations and insulin resistance [27]. Indeed, there has been a worldwide increase in the prevalence of T2DM and NAFLD in concert with the exponential rise in obesity [28]. The current guidelines primarily recommend lifestyle modification and weight loss in obesity, and certain pharmacological treatments in NAFLD patients with T2DM or NASH; however, there are no effective and safe treatments targeting both NAFLD and T2DM [12]. Here, the metformin combination with LD, particularly at higher doses, reduced the HFD-induced weight gains and body fat deposition in the whole body and abdominal region, greater than monotherapies of metformin or LD. The anti-obesity effects were involved in the promotion of the fecal lipid excretion rather than inhibition of the HFD intake. The metformin combination also improved gross aspects of the relevant multi-organs of liver, pancreas, kidney, and fat mass, and the organ weights. Our biochemical and histopathological analyses demonstrated that metformin’s combination with LD can prevent from the development of NAFLD, T2DM, dyslipidemia, and renal steatosis, better than both monotherapies.

Excessive intake of fatty acids leads to hepatic insulin resistance, which reduces the insulin-mediated gluconeogenesis and activates lipogenesis, leading to hepatic triglyceride accumulation as a hallmark of NAFLD [29]. Metformin has previously shown the therapeutic potentials in a HFD-induced NAFLD animal model, and the clinical application actually improves the serum levels of ALT and the partial histological changes [30,31]. Here, the HFD-induced hepatocellular hypertrophy and steatosis were suppressed by the metformin combination with LD, more so than metformin alone, together with reduced levels of blood biochemical parameters for assessing the severity of NAFLD (ALP, ALT, AST, GGT, LDH, glucose, insulin, triglyceride, and total/LDL cholesterol). The combination treatments upregulated AMPK expression, and downregulated ACC1 expression, which may stimulate fatty acid oxidation and inhibit gluconeogenesis and lipogenesis [32,33]. Indeed, the beneficial effects were involved in increased activities of GK responsible for glucose utilization and inhibited activities of G6pase and PEPCK for hepatic gluconeogenesis, suggesting improvement in the insulin resistance and hyperglycemia. Although the glycogen content was not examined here, the data could help to support the hepatic glucose homeostasis. In addition, overflow of free fatty acids (FFA) in hepatocytes saturates mitochondrial β-oxidation, and induces lipid peroxidation and overproduction of reactive oxygen species, leading to cellular damage and inflammation [34]. However, the enhanced antioxidant activities in the combination treatments may contribute to hepatoprotective effects by inhibiting the hyperglycemia-mediated oxidative stress and inflammation in NAFLD [35]. In this context, further clinical studies are needed to clarify the synergic effects in metformin’s combination with LD as a safe traditional phytotherapeutic remedy.

The HFD control showed increased blood glucose, insulin, and HbA1c levels, indicating insulin resistance and hyperglycemia characterized in T2DM [36]. Together with pancreatic islet hyperplasia, the increased number of insulin (β cell-like)-/glucagon (α cell-like)-immunoreactive cells and the ratio of insulin- to glucagon-immunoreactive cells may result from the elevation of insulin secretion to maintain glucose homeostasis under insulin resistance. Metformin is well-known to improve insulin resistance and hyperglycemia by acting as an AMPK activator [7]. Here, the favorable effects of metformin were further enhanced in the combination with LD, probably by improving the altered glucose tolerance and insulin secretory responses of β cells. Furthermore, it is likely that enhanced antioxidant activities of the combination treatments may synergically inhibit glucotoxicity-induced oxidative stress that impairs T2DM [35]. The borders adjacent to the pancreatic islets were distinct from acinar cells with zymogen granules that contain digestive enzymes (i.e., lipase and amylase) [37]. The HFD control showed a reduced number of zymogen granules with acinar atrophy, similar to previous results in animal models of obese T2DM [38,39] and pancreatic steatosis [37,40]. However, the combination treatments increased the exocrine zymogen granules, which could be linked by increased digestion of lipids and other proteins, based on the promoted fecal excretion of triglyceride and total cholesterol [37]. It remains to be elucidated for detailed mechanisms regarding the glucose/insulin tolerance and correlations between lipid metabolism and the other effects, including digestive tract motility.

Chronic exposure to fatty acids leads to fat tissue dysfunction with increased lipolysis and FFA releases, resulting in lipotoxicity-induced insulin resistance in the fat tissue [35]. The exaggerated FFA availability also induces a negative loop in insulin-mediated insulin signaling and glucose utilization in muscle, and further impairs insulin releases in the pancreas [29]. The HFD control showed diabetic dyslipidemia characterized by elevation of serum triglyceride and total/LDL cholesterol levels and a decline of the HDL cholesterol level [41]. The adipocyte hypertrophy/hyperplasia was probably due to a need for accommodating storing the increased lipids. However, the combination treatments further improved the serum levels of triglyceride and cholesterol, and histopathological changes in the fat tissues. The anti-dyslipidemia effects may be involved in the downregulation of adipogenic and lipogenic genes (C/EBP-α/-β, SREBP1, PPARγ, and FAS) and the upregulation of genes exerting transcriptional roles in lipolysis and β-oxidation (PPARα and UCP2) [42]. Furthermore, altered endocrine function of fat tissue develops insulin resistance-related pathogenesis: levels of leptin and adiponectin are associated positively and negatively, respectively, with the severity of NAFLD [43,44]. Leptin stimulates FFA oxidation and glucose uptake, but inhibits lipid accumulation via downregulation of SREBP1 expression, while adiponectin has insulin-sensitizing and anti-inflammatory properties [43]. In this context, the downregulated leptin and upregulated adiponectin in the combination treatments may contribute to improving the imbalanced lipid metabolism and chronic inflammation [3]. The reduced blood parameters (BUN and creatinine) and mild renal steatosis suggest the therapeutic potential of metformin’s combination with LD for metabolic diseases in the multi-organs.

There have been limitations in mono-targeting to the specific pathways in insulin resistance-related metabolic diseases. For example, targeting PPARs has been investigated as an antihyperglycemic medication for treating NAFLD and T2DM; however, the PPARα agonist has only weak effectiveness, or the PPARγ agonist shows undesirable adverse effects, including weight gain [45]. ACC1/2 inhibitors reduce hepatic steatosis, but they have a major concern regarding increased blood lipids [33]. Since the metabolic diseases share a common pathogenesis, including insulin resistance, lipid metabolic alteration, oxidative stress, and inflammation, many studies are also interested in finding α-glucosidase inhibitors and antioxidants among various natural products as the therapeutic sources [46]. Vitamin E, as a potent antioxidant, improves NAFLD with or without T2DM; however, it has little effects to improve hepatic fibrosis, and the long-term use has adverse effects, such as cardiovascular mortality and prostate cancer [47]. There have been some studies reporting greater effects of metformin when combined with leucin, sildenafil, L-cysteine, or atorvastatin for treating NAFLD and T2DM, although data on their clinical efficacy are still lacking [48]. Here, the oral administration of metformin (at 250 mg/kg) combined with LD (more than 100 mg/kg) as a safe, traditional phytotherapeutic remedy showed more favorable effects in treating HFD-induced metabolic alterations than the metformin monotherapy by activating AMPK and lipid metabolism pathways. The clinical oral dose can be speculated as approximately 1.2 g of metformin and 0.49 g of LD (in adults of 60 kg). However, further clinical studies are needed to address the therapeutic efficacy and preventive effects prior to the onset of metabolic diseases.

## 5. Conclusions

These results demonstrated that metformin combined with LD further enhanced the effectiveness of either monotherapies in HFD-induced NAFLD, T2DM, dyslipidemia, and renal steatosis. It suggests that combination of metformin and LD can be a promising therapeutic option for the prevention and treatment of metabolic diseases involving insulin resistance.

## Data Availability

Data is contained within the article and Supplementary Materials.

## Supplementary Materials

The following supporting information can be downloaded at: https://www.mdpi.com/article/10.3390/antiox11030580/s1, Table S1: Primers used for RT-qPCR analysis.

## References

[1] Karczewski J., Sledzinska E., Baturo A., Jonczyk I., Maleszko A., Samborski P., Begier-Krasinska B., Dobrowolska A. (2018). Obesity and inflammation. Eur. Cytokine Netw..

[2] Van Gaal L.F., Mertens I.L., De Block C.E. (2006). Mechanisms linking obesity with cardiovascular disease. Nature.

[3] Tilg H., Moschen A.R. (2006). Adipocytokines: Mediators linking adipose tissue, inflammation and immunity. Nat. Rev. Immunol..

[4] Hernandez E.A., Kahl S., Seelig A., Begovatz P., Irmler M., Kupriyanova Y., Nowotny B., Nowotny P., Herder C., Barosa C. (2017). Acute dietary fat intake initiates alterations in energy metabolism and insulin resistance. J. Clin. Investig..

[5] Younossi Z.M., Golabi P., de Avila L., Paik J.M., Srishord M., Fukui N., Qiu Y., Burns L., Afendy A., Nader F. (2019). The global epidemiology of NAFLD and NASH in patients with type 2 diabetes: A systematic review and meta-analysis. J. Hepatol..

[6] Musso G., Gambino R., Tabibian J.H., Ekstedt M., Kechagias S., Hamaguchi M., Hultcrantz R., Hagstrom H., Yoon S.K., Charatcharoenwitthaya P. (2014). Association of non-alcoholic fatty liver disease with chronic kidney disease: A systematic review and meta-analysis. PLoS Med..

[7] Sanchez-Rangel E., Inzucchi S.E. (2017). Metformin: Clinical use in type 2 diabetes. Diabetologia.

[8] Molavi B., Rassouli N., Bagwe S., Rasouli N. (2007). A review of thiazolidinediones and metformin in the treatment of type 2 diabetes with focus on cardiovascular complications. Vasc. Health Risk Manag..

[9] Makri E., Goulas A., Polyzos S.A. (2021). Epidemiology, Pathogenesis, Diagnosis and Emerging Treatment of Nonalcoholic Fatty Liver Disease. Arch. Med. Res..

[10] Duseja A., Das A., Dhiman R.K., Chawla Y.K., Thumburu K.T., Bhadada S., Bhansali A. (2007). Metformin is effective in achieving biochemical response in patients with nonalcoholic fatty liver disease (NAFLD) not responding to lifestyle interventions. Ann. Hepatol..

[11] Chalasani N., Younossi Z., Lavine J.E., Charlton M., Cusi K., Rinella M., Harrison S.A., Brunt E.M., Sanyal A.J. (2018). The diagnosis and management of nonalcoholic fatty liver disease: Practice guidance from the American Association for the Study of Liver Diseases. Hepatology.

[12] EASL-EASD-EASO (2016). Clinical Practice Guidelines for the management of non-alcoholic fatty liver disease. Diabetologia.

[13] Bolen S., Feldman L., Vassy J., Wilson L., Yeh H.C., Marinopoulos S., Wiley C., Selvin E., Wilson R., Bass E.B. (2007). Systematic review: Comparative effectiveness and safety of oral medications for type 2 diabetes mellitus. Ann. Intern. Med..

[14] Jones G.C., Macklin J.P., Alexander W.D. (2003). Contraindications to the use of metformin. BMJ.

[15] Leoni S., Tovoli F., Napoli L., Serio I., Ferri S., Bolondi L. (2018). Current guidelines for the management of non-alcoholic fatty liver disease: A systematic review with comparative analysis. World J. Gastroenterol..

[16] Schutz K., Carle R., Schieber A. (2006). Taraxacum—A review on its phytochemical and pharmacological profile. J. Ethnopharmacol..

[17] Park C.M., Cha Y.S., Youn H.J., Cho C.W., Song Y.S. (2010). Amelioration of oxidative stress by dandelion extract through CYP2E1 suppression against acute liver injury induced by carbon tetrachloride in Sprague-Dawley rats. Phytother. Res..

[18] Davaatseren M., Hur H.J., Yang H.J., Hwang J.T., Park J.H., Kim H.J., Kim M.J., Kwon D.Y., Sung M.J. (2013). Taraxacum official (dandelion) leaf extract alleviates high-fat diet-induced nonalcoholic fatty liver. Food Chem. Toxicol..

[19] Cho S.Y., Park J.Y., Park E.M., Choi M.S., Lee M.K., Jeon S.M., Jang M.K., Kim M.J., Park Y.B. (2002). Alternation of hepatic antioxidant enzyme activities and lipid profile in streptozotocin-induced diabetic rats by supplementation of dandelion water extract. Clin. Chim. Acta.

[20] Weidner C., Wowro S.J., Freiwald A., Kodelja V., Abdel-Aziz H., Kelber O., Sauer S. (2014). Lemon balm extract causes potent antihyperglycemic and antihyperlipidemic effects in insulin-resistant obese mice. Mol. Nutr. Food Res..

[21] Bolkent S., Yanardag R., Karabulut-Bulan O., Yesilyaprak B. (2005). Protective role of Melissa officinalis L. extract on liver of hyperlipidemic rats: A morphological and biochemical study. J. Ethnopharmacol..

[22] Choi B.R., Cho I.J., Jung S.J., Kim J.K., Park S.M., Lee D.G., Ku S.K., Park K.M. (2020). Lemon balm and dandelion leaf extract synergistically alleviate ethanol-induced hepatotoxicity by enhancing antioxidant and anti-inflammatory activity. J. Food Biochem..

[23] Choi B.R., Cho I.J., Jung S.J., Kim J.K., Lee E.G., Ku S.K., Park K.M. (2021). Lemon Balm and Dandelion Leaf Extracts Synergistically Protect against Carbon Tetrachloride-Induced Acute Liver Injury in Mice. Appl. Sci..

[24] Choi B.R., Kim H.J., Lee Y.J., Ku S.K. (2020). Anti-Diabetic Obesity Effects of Wasabia Japonica Matsum Leaf Extract on 45% Kcal High-Fat Diet-Fed Mice. Nutrients.

[25] Veiga F.M.S., Graus-Nunes F., Rachid T.L., Barreto A.B., Mandarim-de-Lacerda C.A., Souza-Mello V. (2017). Anti-obesogenic effects of WY14643 (PPAR-alpha agonist): Hepatic mitochondrial enhancement and suppressed lipogenic pathway in diet-induced obese mice. Biochimie.

[26] Schmittgen T.D., Livak K.J. (2008). Analyzing real-time PCR data by the comparative CT method. Nat. Protoc..

[27] Liu Z., Zhang Y., Graham S., Wang X., Cai D., Huang M., Pique-Regi R., Dong X.C., Chen Y.E., Willer C. (2020). Causal relationships between NAFLD, T2D and obesity have implications for disease subphenotyping. J. Hepatol..

[28] Panwar B., Hanks L.J., Tanner R.M., Muntner P., Kramer H., McClellan W.M., Warnock D.G., Judd S.E., Gutierrez O.M. (2015). Obesity, metabolic health, and the risk of end-stage renal disease. Kidney Int..

[29] Mu W., Cheng X.F., Liu Y., Lv Q.Z., Liu G.L., Zhang J.G., Li X.Y. (2018). Potential Nexus of Non-alcoholic Fatty Liver Disease and Type 2 Diabetes Mellitus: Insulin Resistance Between Hepatic and Peripheral Tissues. Front. Pharmacol..

[30] Bugianesi E., Gentilcore E., Manini R., Natale S., Vanni E., Villanova N., David E., Rizzetto M., Marchesini G. (2005). A randomized controlled trial of metformin versus vitamin E or prescriptive diet in nonalcoholic fatty liver disease. Am. J. Gastroenterol..

[31] Doycheva I., Loomba R. (2014). Effect of metformin on ballooning degeneration in nonalcoholic steatohepatitis (NASH): When to use metformin in nonalcoholic fatty liver disease (NAFLD). Adv. Ther..

[32] Li R., Chen L.Z., Zhao W., Zhao S.P., Huang X.S. (2016). Metformin ameliorates obesity-associated hypertriglyceridemia in mice partly through the apolipoprotein A5 pathway. Biochem. Biophys. Res. Commun..

[33] Kim C.W., Addy C., Kusunoki J., Anderson N.N., Deja S., Fu X., Burgess S.C., Li C., Ruddy M., Chakravarthy M. (2017). Acetyl CoA Carboxylase Inhibition Reduces Hepatic Steatosis but Elevates Plasma Triglycerides in Mice and Humans: A Bedside to Bench Investigation. Cell Metab..

[34] Polyzos S.A., Kountouras J., Zavos C. (2009). Nonalcoholic fatty liver disease: The pathogenetic roles of insulin resistance and adipocytokines. Curr. Mol. Med..

[35] Roden M., Shulman G.I. (2019). The integrative biology of type 2 diabetes. Nature.

[36] Budde P., Schulte I., Appel A., Neitz S., Kellmann M., Tammen H., Hess R., Rose H. (2005). Peptidomics biomarker discovery in mouse models of obesity and type 2 diabetes. Comb. Chem. High Throughput Screen.

[37] Suttie A.W., Masson R., Schutten M., Suttie A.W. (2018). Chapter 10—Exocrine Pancreas. Boorman’s Pathology of the Rat.

[38] Choi E.H., Chun Y.S., Kim J., Ku S.K., Jeon S., Park T.S., Shim S.M. (2020). Modulating lipid and glucose metabolism by glycosylated kaempferol rich roasted leaves of Lycium chinense via upregulating adiponectin and AMPK activation in obese mice-induced type 2 diabetes. J. Funct. Foods.

[39] Kang S.J., Lee J.E., Lee E.K., Jung D.H., Song C.H., Park S.J., Choi S.H., Han C.H., Ku S.K., Lee Y.J. (2014). Fermentation with Aquilariae Lignum enhances the anti-diabetic activity of green tea in type II diabetic db/db mouse. Nutrients.

[40] Wilson J.S., Korsten M.A., Leo M.A., Lieber C.S. (1988). Combined effects of protein deficiency and chronic ethanol consumption on rat pancreas. Dig. Dis. Sci..

[41] Wu L., Parhofer K.G. (2014). Diabetic dyslipidemia. Metabolism.

[42] Moseti D., Regassa A., Kim W.K. (2016). Molecular Regulation of Adipogenesis and Potential Anti-Adipogenic Bioactive Molecules. Int. J. Mol. Sci..

[43] Tsochatzis E., Papatheodoridis G.V., Archimandritis A.J. (2006). The evolving role of leptin and adiponectin in chronic liver diseases. Am. J. Gastroenterol..

[44] Polyzos S.A., Aronis K.N., Kountouras J., Raptis D.D., Vasiloglou M.F., Mantzoros C.S. (2016). Circulating leptin in non-alcoholic fatty liver disease: A systematic review and meta-analysis. Diabetologia.

[45] Gross B., Pawlak M., Lefebvre P., Staels B. (2017). PPARs in obesity-induced T2DM, dyslipidaemia and NAFLD. Nat. Rev. Endocrinol..

[46] Hays N.P., Galassetti P.R., Coker R.H. (2008). Prevention and treatment of type 2 diabetes: Current role of lifestyle, natural product, and pharmacological interventions. Pharmacol. Ther..

[47] Vilar-Gomez E., Vuppalanchi R., Gawrieh S., Ghabril M., Saxena R., Cummings O.W., Chalasani N. (2020). Vitamin E Improves Transplant-Free Survival and Hepatic Decompensation Among Patients With Nonalcoholic Steatohepatitis and Advanced Fibrosis. Hepatology.

[48] Zhou J., Massey S., Story D., Li L. (2018). Metformin: An Old Drug with New Applications. Int. J. Mol. Sci..

