# Supplementary material for: Combination Effects of Metformin and a Mixture of Lemon Balm and Dandelion on High-Fat Diet-Induced Metabolic Alterations in Mice
Source: Antioxidants (Basel). 2022 Mar 18;11(3):580. doi: 10.3390/antiox11030580 (PMC8945168; doi:10.3390/antiox11030580)
Supplement: Supplementary file 1 [file antioxidants-11-00580-s001.zip › antioxidants-1616124-supplementary.pdf]

Table S1. Primers used for RT-qPCR analysis

Targets (GenBank No.)	Sequence (5' to 3')
ACC1 (NM133360)	Forward: GCCATTGGTATTGGGGCTTAC<br>Reverse: CCCGACCAAGGACTTTGTTG
Adiponectin (NM009605)	Forward: CCCAAGGGAACTTGTGCAGGTTGGATG<br>Reverse: GTTGGTATCATGGTAGAGAAGAAAGCC
AMPKα1 (XM011245321)	Forward: AAGCCGACCCAATGACATCA<br>Reverse: CTTCCTTCGTACACGCAAAT
AMPKα2 (NM178143)	Forward: GATGATGAGGTGGTGGA<br>Reverse: GCCGAGGACAAAGTGC
C/EBPα (NM001287523)	Forward: TGGACAAGAACAGCAACGAGTAC<br>Reverse: CGGTCATTGTCACTGGTCAACT
C/EBPβ (NM001287739)	Forward: AAGCTGAGCGACGAGTACAAGA<br>Reverse: GTCAGCTCCAGCACCTTGTG
FAS (NM007988)	Forward: GCTGCGGAAACTTCAGGAAAT<br>Reverse: AGAGACGTGTCACTCCTGGACTT
Leptin (NM008493)	Forward: CCAAAACCCTCATCAAGACC<br>Reverse: GTCCAACTGTTGAAGAATGTCCC
PPARα (NM011144)	Forward: ATGCCAGTACTGCCGTTTTC<br>Reverse: GGCCTTGACCTTGTTCATGT
PPARγ (NM001127330)	Forward: AGTGGAGACCGCCCAGG<br>Reverse: GCAGCAGGTTGTCTTGGATGT
SREBP1c (XM006532714)	Forward: AGCCTGGCCATCTGTGAGAA<br>Reverse: CAGACTGGTACGGGCCACAA
UCP2 (NM011671)	Forward: CCGCATTGGCCTCTACGACTCT<br>Reverse: CCCCGAAGGCAGAAGTGAAGTG
GAPDH (NM008084)	Forward: CATCTTCCAGGAGCGAGACC<br>Reverse: TCCACCACCCTGTTGCTGTA

ACC1 = acetyl-CoA carboxylase 1, AMPK = 5' adenosine monophosphate-activated protein kinase, C/EBP = CCAAT-enhancer-binding protein, FAS = fatty acid synthase, GAPDH = glyceraldehydes 3-phosphate dehydrogenase, PPAR = peroxisome proliferator-activated receptor, RT-PCR = reverse transcription polymerase chain reaction, SREBP = sterol regulatory element-binding protein, UCP = mitochondrial uncoupling protein.
